# Avoiding sharp accelerations can mitigate the impacts of a Ferry’s radiated noise on the St. Lawrence whales

**DOI:** 10.1038/s41598-022-16060-2

**Published:** 2022-07-15

**Authors:** Dominic Lagrois, Clément Chion, Jean-François Sénécal, Camille Kowalski, Robert Michaud, Valeria Vergara

**Affiliations:** 1 Département des Sciences Naturelles, Université du Québec en Outaouais, Ripon, QC, J0V 1V0 Canada; 2 Groupe de Recherche et d’Éducation sur les Mammifères Marins (GREMM), Tadoussac, QC G0T 2A0 Canada; 3 Raincoast Conservation Foundation, PO Box 2429, Sidney, BC V8L 3Y3 Canada

**Keywords:** Environmental sciences, Ocean sciences

## Abstract

Exposure to anthropogenic noise from the commercial fleet is one of the primary constituents of the acoustic pollution perturbing the environment of aquatic life. Merchant ships (e.g. bulkers, tankers) have been the focus of numerous studies for underwater noise source level determination and modeling. This work extends pre-existing studies to the ferry ship class. Hydrophone-based measurements of the N.M. Trans-Saint-Laurent ferry near the Rivière-du-Loup harbor (Rivière-du-Loup, QC CANADA) were obtained for 186 transits between 2020 July 22th and 2020 September 5th. For each transit, monopole source levels are estimated for two (2) different modes of operation i.e., the low-speed phases of acceleration/deceleration when the ferry launches/docks at Rivière-du-Loup and the passages at quasi-operational speed at the hydrophone’s closest-point-of-approach. Relative differences between the two (2) modes of operation are presented here in the low-frequency domain between 141 and 707 Hz. An average excess of 8 to 11.5 dB indicates that the ferry is likely one order of magnitude noisier, within this frequency band, during acceleration/deceleration when compared to passages at operational speed. This highlights that, in terms of marine mammal conservation, a significant reduction of the noise pollution could be achieved, for instance, by avoiding sudden speed changes in the vicinity of whales.

## Introduction

Vessel underwater noise (VUN) is known to threaten aquatic life^1^ including marine mammals^2^. Impacts of VUN on whales such as belugas include behavioral disruption^3^, changes in vocalizations^4^, masking^5,6^, and hearing loss^7,8^.

Several marine mammal populations that use the St. Lawrence Estuary (SLE) and the Saguenay Fjord (Québec, Canada) are impacted by VUN, including the endangered population of the St. Lawrence Estuary beluga (SLEB) which is protected under the^9^. VUN was identified as one of the three most critical threats limiting SLEB recovery. This led the federal government to develop the Action Plan to reduce the impact of noise on the SLEB and other marine mammals at risk in the SLE^10^.

One of the fundamental goal identified by this action plan is to assess the monopole source levels (MSLs) of all vessels operating in the SLEB’s critical habitat. A ship’s MSLs are equivalent to its far-field frequency-dependent radiated noise corrected for surface reflections also known as Lloyd’s Mirror effects^11^. So far, the estimation of MSLs in the SLE has mainly focused on commercial shipping^12^ and to a lesser extent on whale-watching excursion vessels^13^, with no effort devoted to other segments of the marine traffic. With ferries accounting for the largest cumulative number of transits in the SLEB’s critical habitat^14–16^, this segment definitely deserves more attention with regard to impacts on the St. Lawrence whales’ soundscape.

Beluga whales are considered to be mid- or high-frequency cetaceans^17^. However, the biologically critical contact calls that belugas are known to use for group cohesion and to maintain mother-calf contact contain significant acoustic energy at a broad range of frequencies, including low frequencies^6,18,19^. Moreover, several low-frequency species of baleen whales use the SLE as a feeding ground^20,21^, making them vulnerable to the low-frequency noise emitted by ferries^10^.

Some SLEB’s high-residency areas within their critical habitat are located in shallow waters with depths around 10 m^22,23^. Shallow waters play the role of high-pass filters regarding underwater noise propagation with the lowest transmitted frequency $f_{0}$ given by:

1 $$\begin{aligned} f_{0}\,=\,\frac{c_{w}}{4h\sqrt{1-(c_{w}/c_{b})^2}}, \end{aligned}$$

where $c_{w}$ and $c_{b}$ are respectively the water’s and seabed’s homogeneous speeds of sound and *h*, the depth of the water column. For the shallow Rivière-du-Loup harbor’s seabed consisting in a mixture of clay, silt and sand with $c_{b}/c_{w}$ $\sim$ 1.04–1.06^24^, a significant proportion of low-frequency noise above 150 Hz can still propagate over long distances in a 10-m deep environment. Given the high proportion of the acoustic energy radiated by large vessels below 1000 Hz^15^, the presence of a ferry route in shallow waters is expected to recurrently affect the low-frequency soundscape in this sensitive area, with potential consequences for the belugas that regularly frequent this area.

From May to October, the N.M. Trans-Saint-Laurent makes about 1000 transits through the SLEB’s critical habitat, each of 75 min on average with about 20% of time spent in (full-throttle) acceleration or (reverse-thrust) deceleration. Given the urgent need to reduce VUN in the summer habitat of the endangered SLEB, the overarching goal of this study is to identify avenues to mitigate the noise emitted during ferry transits. In this context, the main objectives of this study were to:

1. Estimate the low-frequency MSLs of the N.M. Trans-Saint-Laurent from multiple recordings made during transits from shallow water measurements;
2. Quantify the impact of the ferry’s acceleration/deceleration on its low-frequency MSLs and compare them with MSLs at a constant quasi-operational speed, and;
3. Investigate the relation between ferry’s speed and its MSL.

Ferries’ operational procedures and practices are identified as high priorities in Recovery Measures 5 and 12 of the Action Plan’s Table 2^10^. Eyesight observations from the N.M. Trans-Saint-Laurent have already confirmed the omnipresence of the SLEB along the SLE’s south shore with peaked numbers of sightings within 1 km of the ferry typically in July and August^25^.

## Material

### Acoustic data

**Figure 1:**
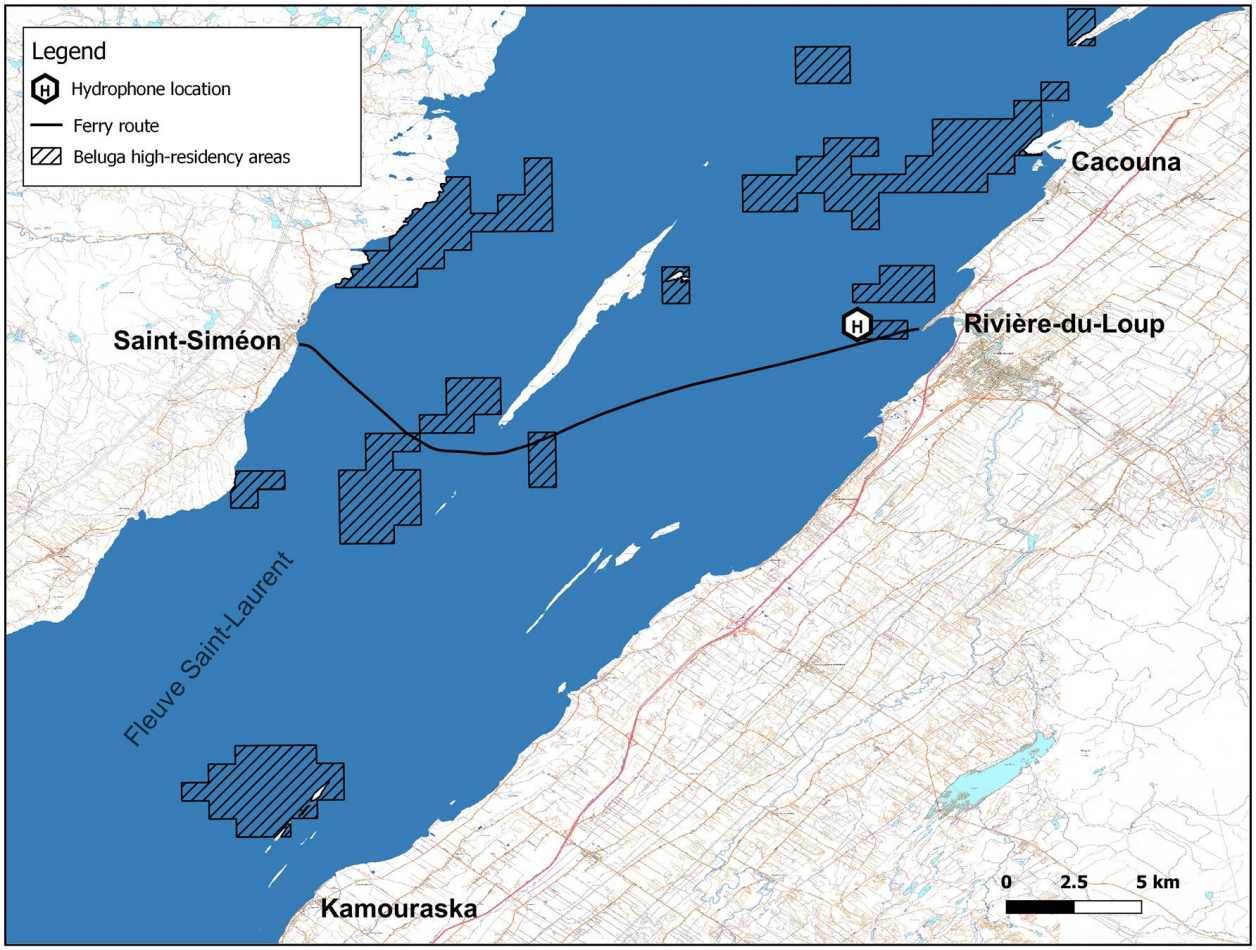
N.M. Trans-Saint-Laurent’s current route between Rivière-du-Loup and Saint-Siméon. The zone of interest where the hydrophone was deployed is located west of the Rivière-du-Loup harbor. Hatched polygons show the SLE beluga’s high-residency areas.

Hydrophone-based measurements were carried out during the summer of 2020 from July 22th to September 5th. A ST300 HF hydrophone (SoundTrap Ocean Instruments, New Zealand) was deployed at $\phi _{0}$ = 47.8499705$^{\circ }$ and $\lambda _{0}\,=\,-\,69.599403^{\circ }$, about 2.23 km from the dock’s western tip outside of the Rivière-du-Loup harbor and a few hundreds of meters north of the transiting routes used by the N.M. Trans-Saint-Laurent ferry to Saint-Siméon on the St. Lawrence river’s north shore (see Fig. 1). An overview of technical specifications of the N.M. Trans-Saint-Laurent is provided in Table 1.

The hydrophone was moored 2 m above seabed. At this position, the Canadian Hydrographic Service gives a water column height $h_{0}$ of 8 m. The hydrophone’s sampling rate was 288 kHz and the end-to-end system sensitivity was − 175.7 dB re 1V $\upmu$Pa$^{-1}$.

**Table 1:** Technical details of the N.M. Trans-Saint-Laurent.

Characteristics (units)	Value
Built year	1963
Length (m)	80
Width (m)	18
Draught (m)	4.2
Operational speed (knots)	12.8
Maximal speed (knots)	15.7
Capacity (vehicles)	100
Capacity (persons)	400
Annual trips per year	~ 1700

### Automatic identification system

Ship positioning data from the Automatic Identification System (AIS) aboard the ferry was obtained from Parks Canada. The AIS system is an autonomous tracking system that provides GPS positions at frequent intervals as well as some information about the ship including speed and direction. AIS data are regularly used in conservation science^26^. The AIS data of the ferry was extracted during the study period and the timestamps were converted to local time.

For each AIS entry, the ferry’s distance to the hydrophone (*d*) and its instantaneous speed-through-water (STW) were computed using respectively the ferry’s position ($\phi$,$\lambda$), and its course over ground (COG), speed-over-ground (SOG) and the vector of surface currents orientation and speed from the closest prediction from hourly models at an approximate 250-m resolution (https://ogsl.ca)^27,28^.

During the period of interest, the N.M. Trans-Saint-Laurent ferry was scheduled for six (6) trips a day i.e., three (out)going to Saint-Siméon from Rivière-du-Loup (with departures at 08:00 EDT, 12:00 EDT, and 16:00 EDT) and three (in)coming from Saint-Siméon to Rivière-du-Loup (with arrivals approximately scheduled at 11:00 EDT, 15:00 EDT, and 19:00 EDT).

## Methods

### Bandwidth of interest

Details regarding the processing of the post-retrieval data are provided in “Appendix 1”. Sound pressure levels (SPLs), hereafter referred to as the frequency-dependent received noise levels (RLs) at the hydrophone, were extracted using Matlab$^{\circledR }$-supported PAMGuide^29^. RLs spectra were processed for a low-frequency bandwidth between $f_{0}$ = 141 Hz (see Eq. 3) and $f_{1}$ = 707 Hz (see details in “Appendix 1”). The lower and upper limits $f_{0}$ and $f_{1}$ are respectively attributed to the natural high-pass filter caused by the shallow-depth environment where measurements took place and signal contamination at mid-to-high frequencies (> 1 kHz).

### Backpropagation and MSLs calculations

Details on the computation of source noise levels (SLs) are provided in “Appendix 2”. The passive SONAR equation (see Eq. 6) is used to process SLs spectra between $f_{0}$ and $f_{1}$ by adding, frequency by frequency, RLs to the propagation loss sustained by the sound wave between the ferry and the hydrophone’s position. Considering the shallow-depth and low-frequency domains characterizing this work, the parabolic-equation solver RAM^30^ was used to predict sound attenuation along the lines-of-sight connecting the ferry to the hydrophone^31,^ with hydrometric (Observatoire global du Saint-Laurent) and geological^32^ input data referenced therein.

Integrated in the frequency domain between $f_{0}$ and $f_{1}$, RLs and SLs spectra provide respectively broadband received levels BB$_{\text {RL}}[f_{0}-f_{1}$] (see Eq. 4) and broadband source levels BB$_{\text {MSL}}[f_{0}-f_{1}$] (see Eq. 7).

The acoustic impact of the ferry’s acceleration/deceleration was assessed by comparing BB$_{\text {MSL}}$[$f_{0}-f_{1}$] values at quasi-operational speed usually happening at closest-point-of-approach (CPA) with BB$_{\text {MSL}}[f_{0}-f_{1}$] values calculated for episodes of sharp acceleration/deceleration variations at launching/docking in Rivière-du-Loup harbour (Fig. 1).

### Generalized linear mixed model

A multi-parameter maximum likelihood approach via the minimization of the Akaike information criterion was used to assess the dependency between the ferry’s MSLs and its speed with emphasis on transits at CPA where recorded speeds in this work compare with the ferry’s operational speed (see Table 1). Generalized linear mixed model (GLMM) analysis was conducted with the function *lmer* of the *lme4* package^33^. The term “mixed” indicates that the model implies the use of at least one fixed effect (i.e., a variable for which we wish to quantify the effect on reported broadband source levels) and at least one random effect (in our case, the time of the day of the measurement). Confidence intervals and *p*-values (via Wald-statistics approximation) were calculated with the function *sjt.lmer* of the *sjPlot* package^34^.

### Definitions

The following concepts are largely discussed in the next sections:

- Acceleration phase:
         *The 10-to-15 min time period required by the ferry to reach its operational speed following departure, at rest, from the Rivière-du-Loup dock.*
- Deceleration phase:
         *The 10-to-15 min time period used by the ferry to lower its speed from operational to full stop in its final approach at the Rivière-du-Loup dock.*

These definitions also certainly apply to the Saint-Siméon dock although the required time periods for both phases can always vary depending on the sea state, crowding in the harbor, specific bathymetric features (e.g., ref.^35^), and more.

## Results

### Ferry’s MSL

**Figure 2:**
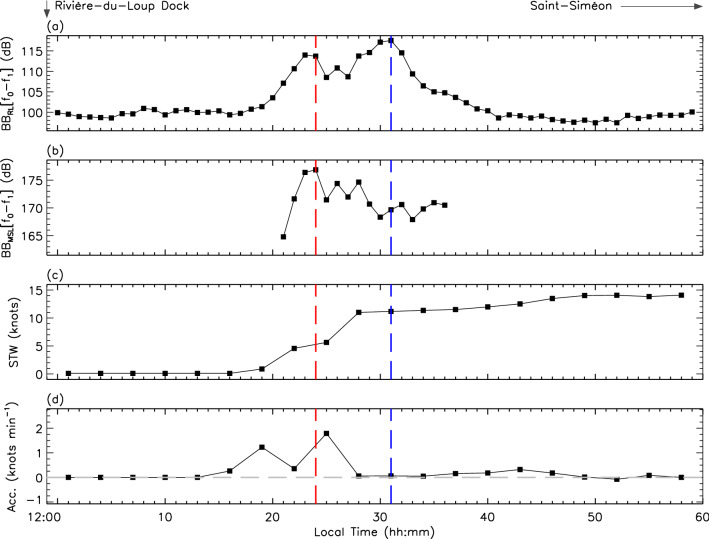
Ferry’s departure on 2020-08-05 12:00-to-13:00 EDT. The dock is located on the far-left of the figure. The ferry moves from left to right towards Saint-Siméon positioned on the figure’s far-right. (**a**) Amplitude of the frequency-integrated received noise levels (BB$_{\text {RL}}$[$f_{0}-f_{1}$]) as a function of the local time. Integration along the frequency axis is carried out between $f_{0}$ and $f_{1}$ (see text). (**b**) Predictions of the frequency-integrated monopole sourve levels (BB$_{\text {MSL}}$[$f_{0}-f_{1}$]) as a function of the local time. (**c**) AIS’s speed-through-water (STW). (**d**) Time derivative of the speed-through-water curve shown in (**c**). Accelerations are displayed in units of knots min$^{-1}$. To compensate for any desynchronisation between the hydrophone’s internal clock and the AIS timestamp, profiles in (**c**) and (**d**) were smoothed using a 3-min boxcar function. The blue vertical line indicates the time at which the closest-point-of-approach (CPA) to the hydrophone was reached and the red, the time of the highest BB$_{\text {MSL}}$[$f_{0}-f_{1}$] prediction. The sign of the instantaneous acceleration (i.e., whether STW is increasing or receding) is shown by the gray horizontal line in (**d**).

Figure 2 displays results obtained from a typical case of an outgoing trip towards Saint-Siméon. The ferry leaves Rivière-du-Loup at approximately 12:16 EDT, roughly 15 min late on its original schedule. Between 12:17 EDT and 12:31 EDT, the ship enters an acceleration phase towards its operational speed (see Table 1). The acceleration phase is always carried out in two (2) distinct steps as revealed by the double-peak signature of the acceleration profile, seen at 12:19 EDT and 12:25 EDT in the lower panel. This may suggest two (2) distinct modes of operation of the engines, the first to break the ship’s inertia and the second to reach operational speed. By the time the ferry transits at CPA at 12:31 EDT, operational speed has been approximately reached and the acceleration declines towards 0 knot min$^{-1}$. The BB$_{\text {RL}}$[$f_{0}-f_{1}$] profile shows that the highest noise levels recorded at the hydrophone coincide with the CPA position, where the source-to-hydrophone distance is minimal. Relatively high-amplitude features are also detected preceding CPA during the 14 min of the acceleration phase. Backpropagation of the RLs spectra indicates that the peak BB$_{\text {MSL}}$[$f_{0}-f_{1}$] happened at 12:24 EDT when the ferry’s STW was about 5 knots and climbing. Increase of the engines’ regime during these moments of acceleration certainly contributes to the radiation of substantial underwater low-frequency noise on distances greater than 1 km.

**Figure 3:**
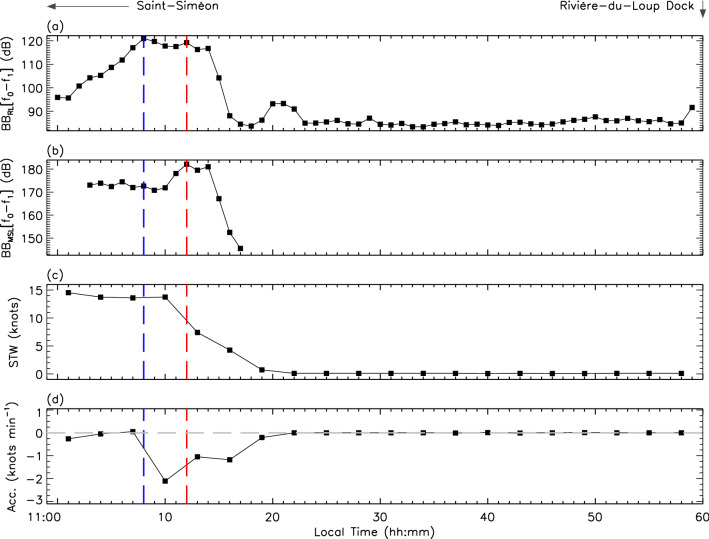
Same as Fig. 2 for the ferry’s arrival on 2020-07-22 11:00-to-12:00 EDT. Saint-Siméon is located on the far-left of the figure. The ferry moves from left to right towards the dock positioned on the figure’s far-right.

Figure 3 reveals similar trends for an incoming trip towards Rivière-du-Loup. Once the ferry has crossed CPA while traveling at quasi-operational speed at 11:08 EDT, a deceleration phase is initiated between 11:09 EDT and 11:19 EDT as it approaches the dock. An inverse double-peak signature characterizes the deceleration profile again pointing towards a change in the engines’ regime during the ship’s docking procedure. BB$_{\text {RL}}$[$f_{0}-f_{1}$] measurements are highest at CPA but persisted with similar amplitude values well into the deceleration phase. Backpropagated BB$_{\text {MSL}}$[$f_{0}-f_{1}$] suggested that the ferry is definitely noisier while decelerating towards its docking approach when compared to the radiated noise at near operational speed. This indicates that the ship’s engines could be on a reverse-thrust mode rather than simply gliding in its docking approach.

Time periods of interest are identified here between the acceleration kickoff and the passage at CPA for outgoing trips, and the passage at CPA and the end of the deceleration downgrade for incoming trips. Table 5 displays the specific results for the CPA and acceleration/deceleration measurements. At CPA, local time ($t_{\text {CPA}}$), ferry-to-hydrophone distance ($d_{\text {CPA}}$), ferry’s speed-through-water (STW$_{\text {CPA}}$) and acceleration ($a_{\text {CPA}}$), and calculated broadband source levels (BB$_{\text {MSL}}^{\text {CPA}}$[$f_{0}-f_{1}$]) are provided. The local time of a given acceleration/deceleration event ($t_{acc}$) was defined as the inflection point (i.e., change of sign of the jerk profile, d$^{3}\vec {r}$/d$t^{3}$) of the acceleration profile between the two (2) identified peaks (e.g., 12:22 EDT in Fig. 2d and 11:13 EDT in Fig. 3d). This favors the probability that source-level measurements, during the acceleration/deceleration phase, are gathered for the ferry operating in similar mechanical conditions from one transit to another. At $t_{acc}$, Table 5 provides the ferry-to-hydrophone distance ($d_{acc}$), ferry’s speed-through-water (STW$_{acc}$) and acceleration ($a_{acc}$) at jerk’s sign change, and calculated broadband source levels (BB$_{\text {MSL}}^{acc}$[$f_{0}-f_{1}$]). Missing data in our AIS-based spreadsheet between 2020 August 11th and August 24th, and the fact that ferry was temporarily disabled for maintenance on 2020 August 30th prevent the use of these recordings in Table 5.

**Figure 4:**
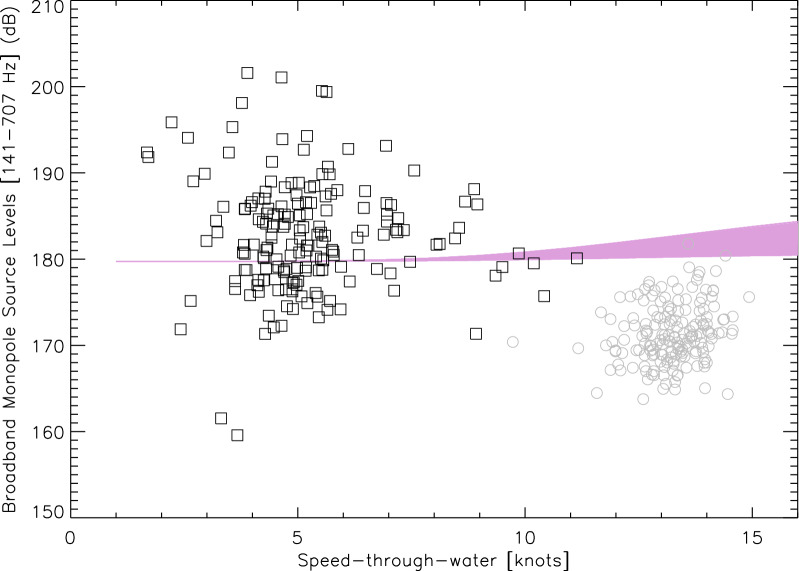
Speed-through-water (STW$_{\text {CPA}}$, STW$_{acc}$) vs. broadband source levels ($\text{BB}_{\text {MSL}}^{\text {CPA}}$[$f_{0}-f_{1}$], BB$_{\text {MSL}}^{acc}$[$f_{0}-f_{1}$] ) according to Table 5. Gray and black data respectively correspond to measurements at $t_{\text {CPA}}$ and $t_{acc}$. Broadband monopole source levels integrated between $f_{0}$ and $f_{1}$ as predicted by^36^ for merchant ships are provided in purple. The model’s space parameters used here include ship’s length (150–250 m), width (28–44 m), draught (6–14 m), engine’s mass (50–250 t.), and number of engines in operation at the same time (1–5).

Figure 4 shows how broadband source levels at $t_{\text {CPA}}$ and $t_{acc}$ behave with respect to the ferry’s STW. For comparison, the Wittekind's^36^ MSL models for merchant ships is also displayed.

### CPA versus acceleration/deceleration

**Figure 5:**
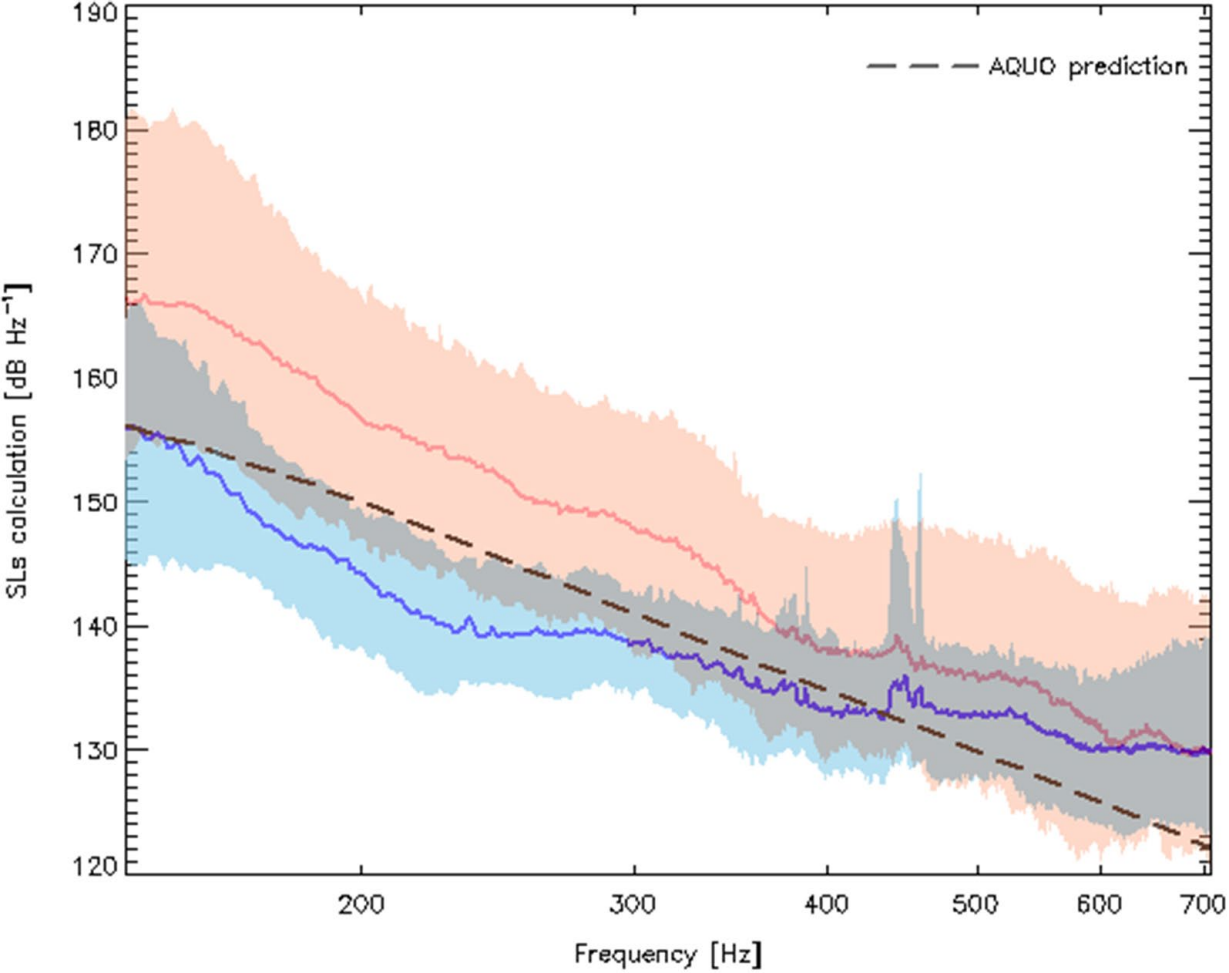
Distributions of the backpropagated SLs spectra obtained during the acceleration/deceleration phases (red) and during CPA transits (blue) between $f_{0}$ and $f_{1}$. Median spectra are shown as thick colored lines while 5% and 95% envelops are displayed in light colors. The black long-dashed line shows the SLs prediction for the ferry class of the surface-corrected Audoly's^41^ model (see the authors’ $\S$ 5.4) for a ship’s length of 80 m and traveling at an operational speed of 12.8 knots (see Table 1).

Figure 5 shows the comparison between median SLs spectra predicted at $t_{\text {CPA}}$ and $t_{acc}$ (see “Ferry’s MSL” section) between $f_{0}$ and $f_{1}$. At CPA, when the ferry usually travels at a quasi-operational speed (see Table 1), the agreement with the ferry model developed by^37^ gives credence to our results.

**Figure 6:**
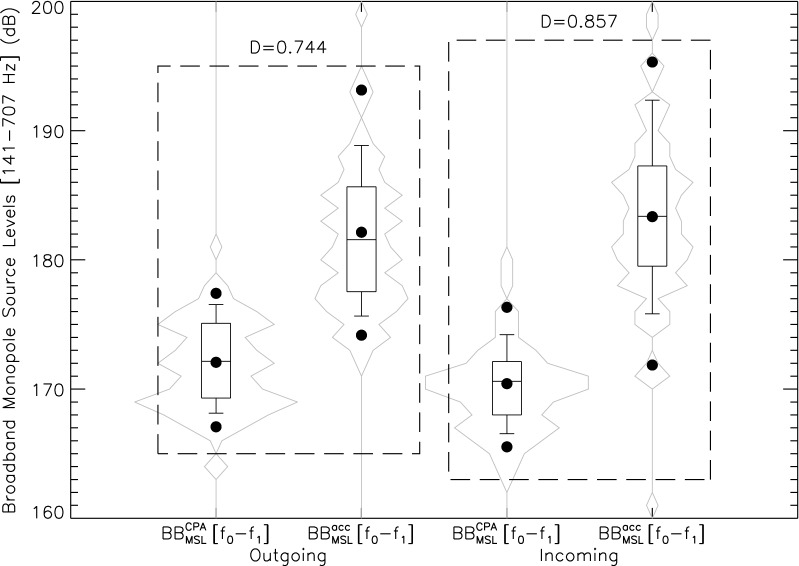
Whisker plots of the BB$_{\text {MSL}}^{\text {CPA}}$[$f_{0}-f_{1}$] and BB$_{\text {MSL}}^{acc}$[$f_{0}-f_{1}$] distributions provided in Table 5. Percentiles are shown, from bottom to top, at 5% (dot), 10%, 25%, 50%, 75%, 90%, and 95% (dot). The central dot gives the mean of the distribution. Violin plots tracing out the shape of each distributions are added. The KS-statistic *D* between CPA and acceleration/deceleration measurements is fairly close to 1 for both outgoing and incoming trips, hence suggesting a genuine statistical difference in radiated source noise levels between both operational modes.

Average BB$_{\text {MSL}}^{\text {CPA}}$[$f_{0}-f_{1}$] at quasi-operational speed are estimated at 172.07 ± 3.42(1$\sigma$) dB and 170.42 ± 3.27(1$\sigma$) dB respectively for outgoing and incoming trips. By comparison, average BB$_{\text {MSL}}^{acc}$[$f_{0}-f_{1}$] values of 182.14 ± 5.82(1$\sigma$) dB and 183.34 ± 7.12(1$\sigma$) dB are respectively found for outgoing and incoming trips during the acceleration/deceleration phase. Whisker plots are provided in Fig. 6. Table 2 shows the trips’ time division for typical days during the summer of 2020. The combined acceleration/deceleration phases account for roughly 20% the time required to complete a given trip. This suggests that the ship’s source-level signature cannot be modeled using a constant operational speed approximation and that low-speed launching and docking events must be considered in the overall characterization of its acoustic impact.

**Figure 7:**
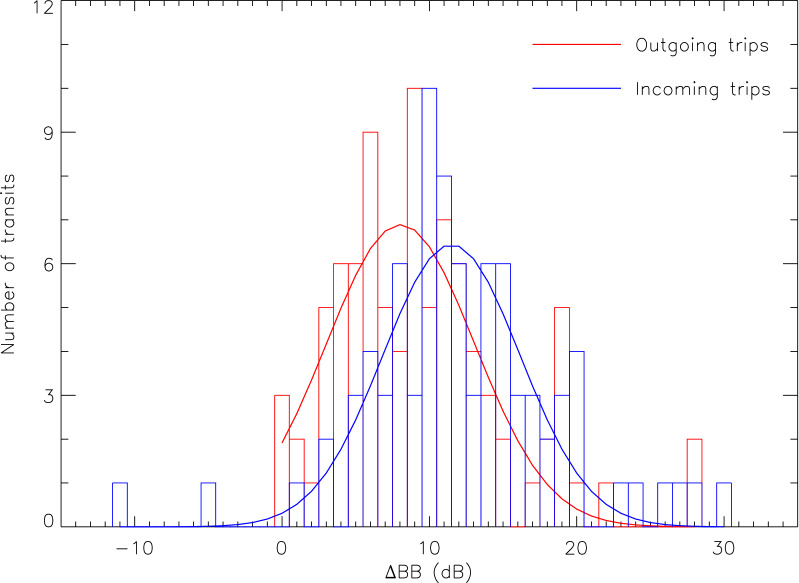
Histograms of BB$_{\text {MSL}}^{acc}$[$f_{0}-f_{1}$] - BB$_{\text {MSL}}^{\text {CPA}}$[$f_{0}-f_{1}$] according to the data listed in Table 5. Outgoing (in red) and incoming (in blue) ships are treated separately. Solid lines are the corresponding normal distributions fitted on each histogram.

Given the results for the Kolmogorov–Smirnov statistics in Figs. 6 and 7 displays the $\Delta$BB $\equiv$ BB$_{\text {MSL}}^{acc}$[$f_{0}-f_{1}]\,-\,BB_{\text {MSL}}^{\text {CPA}}$[$f_{0}-f_{1}$] distributions for both outgoing and incoming trips treated separately. Assuming normal distributions, $\Delta$BB is centered on 8.04 ± 5.02(1$\sigma$) dB for outgoing trips and 11.50 ± 4.67(1$\sigma$) dB for incoming trips.

### Effects of the Ferry’s speed and acceleration on MSL

Ferry-to-hydrophone distances ($d_{\text {CPA}}$, $d_{acc}$), speeds-through-water (STW$_{\text {CPA}}$, STW$_{acc}$), and accelerations ($a_{\text {CPA}}$, $a_{acc}$) were treated as fixed effects in the GLMM statistics (see “Generalized linear mixed model” section). Results, shown in Table 3, suggest a correlation (*p*-values = [0.001–0.022]) between STW$_{\text {CPA}}$ and BB$_{\text {MSL}}^{\text {CPA}}$[$f_{0}-f_{1}$] estimated at 1.41 and 1.49 dB knot$^{-1}$ respectively for outgoing and incoming trips. No correlation could be established between the magnitude of the ferry’s acceleration and the corresponding broadband source levels.

We verified that the fit of each of the four (4) models shown in Table 3 was good with the R package *DHARMa* version 0.4.5^38^.

## Discussion

Table 5 shows broadband MSLs, frequency-integrated between 141 and 707 Hz, averaging at 171.28 ± 3.44(1$\sigma$) dB and 182.72 ± 6.49(1$\sigma$) dB respectively at $t_{\text {CPA}}$ for the CPA transits at quasi-operational speed and at $t_{acc}$ during the acceleration/deceleration phases close to the dock. Although uncertainties on the sediments’ parameters (see “Appendix 2”) raise concerns regarding comparisons with other studies, values obtained here for the noisy acceleration/deceleration mode are comparable with predictions of^36^, across the same frequency band, for merchant ships (see Fig. 4). This suggests that the low-frequency acoustical footprint of an accelerating ferry could be similar to the one of much larger tankers and bulkers traveling at operational speed (although a comparison between our MSLs results and results/predictions for other ships should be nuanced because of protocol’s limitations; see “Discussion” section).

Figures 4, 5, and 6 reveal that the ferry radiates more noise during the low-speed acceleration/deceleration phases than during its design-speed operational mode. This is contrary to most SLs empirical models that usually propose a monotonically increasing trend of the radiated noise levels with traveling speed (e.g.,^39–41^). Since these models were almost all exclusively developed for quasi-operational speed transits, acceleration/deceleration has never been seen as a suitable predictor in MSLs modeling. Our results hence suggest that current MSLs models might underestimate the underwater noise radiated by ships during speed change phases, which could lead to non marginal errors for vessels that spend significant time in acceleration/deceleration modes such as ferries. This reinforces our assumption that conditions in whale habitats in the vicinity of busy harbors could be improved by limiting sharp accelerations/decelerations from vessels.

In Fig. 7, the correlation with direction could be an indication of the ferry’s anisotropic radiated sound field with higher noise levels being emitted at the stern close to the engines and propellers. This is geometrically plausible given azimuthal angles of view differing by $\cos ^{-1}({d_{\text {CPA}}/d_{acc}})$ (roughly 65$^{\circ }$) between $t_{\text {CPA}}$ and $t_{acc}$ (see Table 5). Considering the time periods of interest defined in “Ferry’s MSL” section, the stern of the ship faces the hydrophone only during the deceleration phase of incoming trips. This can also be seen in Fig. 6 where BB$_{\text {MSL}}^{acc}$[$f_{0}-f_{1}$] is slightly greater, on average 1.3 dB, for decelerated-incoming trips when compared to accelerated-outgoing trips. Figure 6 also reveals a side-to-side asymmetry at CPA resulting in BB$_{\text {MSL}}^{\text {CPA}}$[$f_{0}-f_{1}$] being on average about 2 dB louder as seen from a port aspect (outgoing trips) when compared to starboard measurements (incoming trips). This agrees with the port/starboard noise directivity recorded for cargos (about 2–3 dB at *f* $=$ 340–360 Hz)^42^ and containers (< 9 dB for *f* < 50 Hz)^43^.

**Table 2:** Time division of standard N.M. Trans-Saint-Laurent’s Trips.

Date	Departure (EDT)	Duration (min)	Acceleration (launching) (min)	Design speed (min)	Deceleration (docking) (min)	% of time in acceleration/deceleration
2020-07-22	08:02	89	10	71	8	20.2
2020-07-22	12:02	82	8	67	7	18.3
2020-07-22	16:01	75	8	59	8	21.3
2020-07-23	08:02	81	8	65	8	19.8
2020-07-23	12:09	80	7	65	8	18.8
2020-07-23	16:05	70	7	55	8	21.4
2020-07-24	08:13	79	8	62	9	21.5
2020-07-24	12:13	81	9	64	8	21.0
2020-07-24	16:09	71	8	53	10	25.4
2020-07-25	08:08	76	8	58	10	23.7
2020-07-25	12:01	83	9	64	10	22.9
2020-07-25	16:01	74	8	56	10	24.3
2020-07-22	10:07	73	7	57	9	21.9
2020-07-22	14:12	80	4	70	6	12.5
2020-07-22	17:38	74	4	61	9	17.6
2020-07-23	09:58	68	7	51	10	25.0
2020-07-23	14:16	75	4	64	7	14.7
2020-07-23	17:40	69	4	58	7	15.9
2020-07-24	10:21	72	5	56	11	22.2
2020-07-24	14:20	78	5	64	9	17.9
2020-07-24	18:01	73	4	62	7	15.1
2020-07-25	09:56	67	6	51	10	23.9
2020-07-25	14:13	75	7	60	8	20.0
2020-07-25	17:33	75	4	63	8	16.0

**Table 3:** Generalized linear mixed models.

Predictor	Outgoing trips			Incoming trips		
	Estimate	Confidence interval	*p*-value	Estimate	Confidence interval	*p*-value
**BB**$_{\mathbf {MSL}}^{\mathbf {CPA}}$[${\varvec{f}}_{\varvec{0}}-{\varvec{f}}_{\varvec{1}}$] **(dB)**						
Intercept	149.18	134.73 to 163.62	**< 0.001**	146.29	129.12 to 163.47	**< 0.001**
$d_{\text {CPA}}$	4.99	− 0.45 to 10.43	0.072	4.51	− 8.69 to 17.71	0.503
STW$_{\text {CPA}}$	1.41	0.21 to 2.62	**0.022**	1.49	0.57 to 2.41	**0.001**
$a_{\text {CPA}}$	1.80	− 3.88 to 7.48	0.534	− 0.79	− 2.32 to 0.75	0.315
**BB**$_{\mathbf {MSL}}^{{\varvec{acc}}}$[${\varvec{f}}_{\varvec{0}}-{\varvec{f}}_{\varvec{1}}$] (**dB**)						
Intercept	133.74	93.47 to 174.01	**< 0.001**	212.99	182.51 to 243.47	**< 0.001**
$d_{acc}$	19.20	− 0.36 to 38.75	0.054	− 11.73	− 25.59 to 2.13	0.097
STW$_{acc}$	1.84	0.31 to 3.37	0.068	− 1.81	− 3.48 to − 0.14	0.054
$a_{acc}$	0.65	− 3.86 to 5.17	0.776	− 1.15	− 8.74 to 6.43	0.765

The null hypothesis can be rejected for *p*-values shown in bold.

Correlation between source-level measurements and corresponding STW values at CPA is suggested in Table 3 with slopes of 1.41 and 1.49 dB knot$^{-1}$, strongly agreeing with the 1 dB knot$^{-1}$ value commonly reported in the literature for merchant ships^44–46^. These studies provide their results on typically much larger bandwidths than what was used in this work. Hence, it is reasonable to assume that the acoustical gain in dB per knot would exceed values reported here if the full bandwidth of the SLEB’s audiogram (i.e., $\sim$ 150–150,000 Hz) would have been considered. The CPA configuration in this work is similar to what would have been used in the determination of MSLs empirical laws in the literature (e.g.,^36,39,41,47^). Hence, a correlation between source-levels and transiting speed is not surprising, although the relatively narrow distribution of the STW$_{\text {CPA}}$ values in Table 5 makes it difficult to properly assess the impact of speed on BB$_{\text {MSL}}^{\text {CPA}}$[$f_{0}-f_{1}$].

Accelerations at CPA are always close to 0 since, at this point, the ship always travels at relatively constant speed and, therefore, should have little impact on source-level measurements. We also found no direct correlation between $a_{acc}$ and broadband source levels BB$_{\text {MSL}}^{acc}$[$f_{0}-f_{1}$] retrieved from the acceleration/deceleration phases. As expected, source-level measurements are geometry independent and no correlation was found with the source-to-hydrophone distances *d*, hence giving credence to the backpropagation algorithm in this range of frequencies (see “Appendix 2”). We suspect that a better correlation between source-level measurements and the ferry’s engine’s RPM^48^ may have been found although RPM values could not be retrieved *a posteriori* for this study.

Sources of uncertainties on values for BB$_{\text {MSL}}^{\text {CPA}}$[$f_{0}-f_{1}$] and BB$_{\text {MSL}}^{acc}$[$f_{0}-f_{1}$] listed in Table 5 are summarized as follow.

The 100-m mesh grid of the bathymetric data (see “Appendix 2”) is the limiting factor in terms of spatial resolution. This could be of importance in the case of close ship-to-hydrophone interactions separated by a few hundreds meters (see $d_{\text {CPA}}$ in Table 5) if localized bathymetric irregularities of restrained dimensions are found along the line-of-sight connecting the source and the receiver.

The AIS data (see “Automatic identification system” section) requires our analysis to be carried out in averaged blocks of 1 min. For quasi-operational speed passages at CPA, we expect the ship’s radiated noise to remain roughly constant. However, this can not be said for the acceleration/deceleration phase in which speed and likely engines’ regime change quickly. High-amplitude spikes of change in the radiated noise levels could be lost in the averaging statistics.

The impacts of meteorological conditions were not considered. The wind’s magnitude and orientation will have an impact on the release of energy required by the engines to maintain (or accelerate to) a given speed^48^. Sea conditions, that could add to the levels of noise received by the hydrophone, were also ignored (see Fig. 14 of^49^). The geophysical parameters of the terrain sediments are also questioned given the dated low-resolution reference^32^ used to quantify them in this work (see “Appendix 2”).

**Figure 8:**
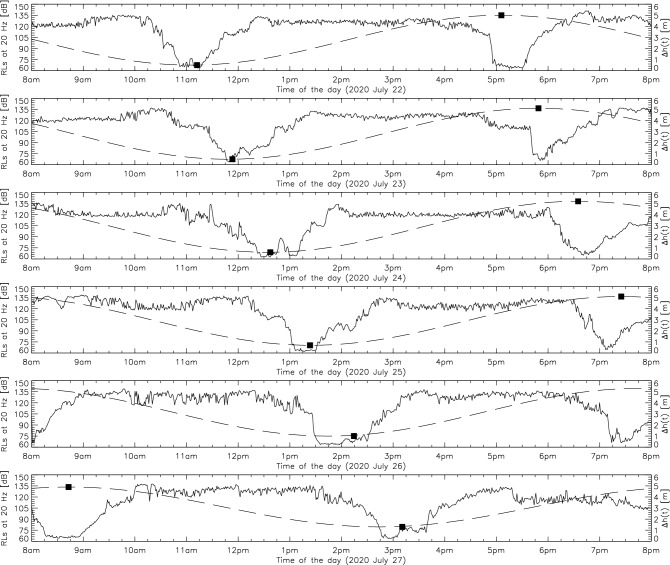
Time series of the RLs measurements at 20 Hz retrieved from the Kamouraska quiet coastal island (see Fig. 1). The recording setup and data processing methods are identical to the description in “Acoustic data” section. Curves of received noise levels are shown for full 12-h periods of continuous recording. No AIS-listed ships were present. Filled squares indicate the local EDT time of the day for low and high tides (see right-hand ordinate). Sinusoidal fits of the ebb and flood tides are shown as long-dashed lines.

The absence of ISO/ANSI standard measurements (for instance: shallow waters) limits our ability to isolate the contribution of environmental variables. Ainslie et al.^49^ discussed the formal bases of a standard approach for the measurement of underwater radiated noise by vessels transiting in shallow waters (*z* < 30m). The authors in particular suggested the use of at least three (3) hydrophones deployed directly on the seabed in order to smooth out pseudo-noise effects caused by current flows. Figure 8 shows the synchronicity between the measured ambient noise and the tidal diurnal pattern in the neighboring quiet coastal island of Kamouraska. Quiet periods are correlated with the peaked low/high tides while currents engulf the hydrophone causing turbulent vortices and pseudo-noise during episodes of flood/ebb tides.

These limits would have been of concern if part of our objectives was to estimate absolute (as opposed to relative) MSLs values for the N. M. Trans-Saint-Laurent. However, this work mainly focuses on identifying mitigation avenues to reduce the ferry’s acoustical footprint on a sensitive area frequented by the SLEB. All MSLs measurements are relative to the same ship transiting at the same location. Hence, it is reasonable to assume that uncertainties on MSLs values attributed to protocol’s limitations do not impact the conclusions reached in this work.

## Conclusion

A hydrophone deployment close ($\lesssim$ 2 km) to the current route of the N. M. Trans-Saint-Laurent ferry allowed to estimate the ship’s monopole source levels, frequency-integrated between 141 and 707 Hz, for different engines’ regime. Phases of acceleration as the ferry launches from the nearby Rivière-du-Loup harbor, of deceleration as the ferry prepares to dock in the Rivière-du-Loup harbor, and of roughly constant quasi-operational speed as the ferry transits at the hydrophone’s closest-point-of-approach were prioritized.

This work is highlighted by the large numbers of transits (186) recorded for a single ship as opposed to other studies dedicated to ships’ source-level characterisation that usually imply a much lower number of transits of the same ship or many transits but of different ships.

The main results of our study are:

1. A correlation of approximately 1.4 dB knot$^{-1}$ was established between the ferry’s speed-through-water in a quasi-operational mode at CPA and its monopole source levels. No other correlation could be determined between the computed monopole source levels and the ship’s kinematical properties and sea conditions at the time of the recordings. This highlights the requirement of new standards for shallow water recordings in order to properly assess the role of environmental variables in the extraction of monopole source levels.
2. Results have shown that the ferry could be one order of magnitude ($\gtrsim$ 10 dB) noisier during sharp speed changes when compared to the constant design speed used at operational mode. Quick accelerations and decelerations likely impact the levels of low-frequency radiated noise on kilometers away from the source. This work provides an additional approach to mitigate underwater noise pollution by regulating, if possible, the engines’ regime when ships and noise-sensitive marine mammals are in close encounters.
3. Anisotropy in the radiated noise field has been confirmed as a mean difference of 1.3 dB in monopole source levels was found in favor of stern-oriented recordings i.e., when engines and propellers faced the hydrophone. A port/starboard asymmetry of nearly 2 dB was also found as the ferry transited at CPA.
4. Given the lack of comparative studies in the literature, we cannot conclude if the results presented here are representative of the world’s ferry fleet although it is not surprising that phases of acceleration/deceleration radiate more noise than transits at operational speed. To generalize this result, other *ad hoc* recordings of different ferries operating in ecologically sensitive habitats are required.

This work is, to our knowledge, the first to establish a causal relationship between a ship’s acceleration and its underwater radiated noise. Two (2) mitigation avenues are proposed in order to restrain the acoustic disturbance of ferries in areas populated by noise-sensitive species:

1. Avoid episodes of sharp full-throttle acceleration or reverse-thrust deceleration, and;
2. Smoothly slow down by reducing RPM when possible and safe to do so, complying with the good seamanship practices.

Results, shown in this work, will be thoroughly investigated and generalized to the whole fleet of ferries currently active in the St. Lawrence Estuary beluga’s habitat. Standardized, hydrophone-based deployments for environments of intermediate depths are scheduled to take place in the spring of 2023. Accelerations, speeds-through-water, and RPM will act as controlled parameters as the ferries’ underwater radiated noise will be recorded during voluntary passages.

## Figures and Tables

**Figure 9:**
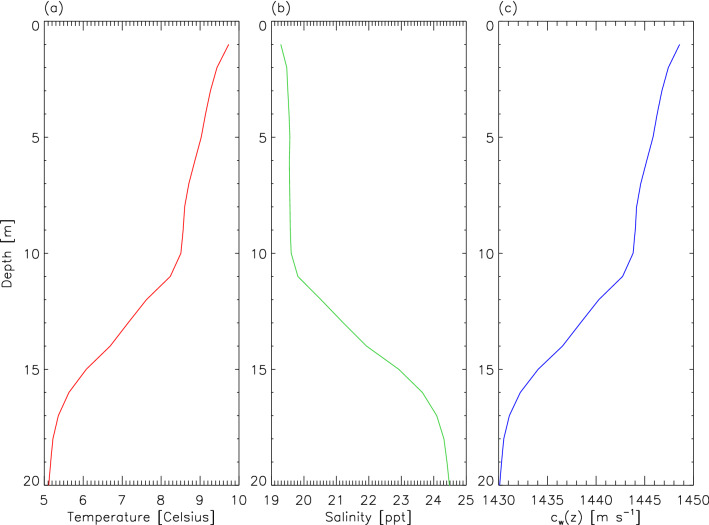
(**a**) Average water temperature profile. (**b**) Average water salinity profile. (**c**) Corresponding average speed of sound profile. CTD data were retrieved from the Observatoire global du Saint-Laurent archives and were gathered from June to August between 2006 and 2010. Most of the data were gathered off the Kamouraska harbor (see Fig. 1). The speed of sound profile is dominated by the drop in temperatures measured at depths greater than 10 m.

**Figure 10:**
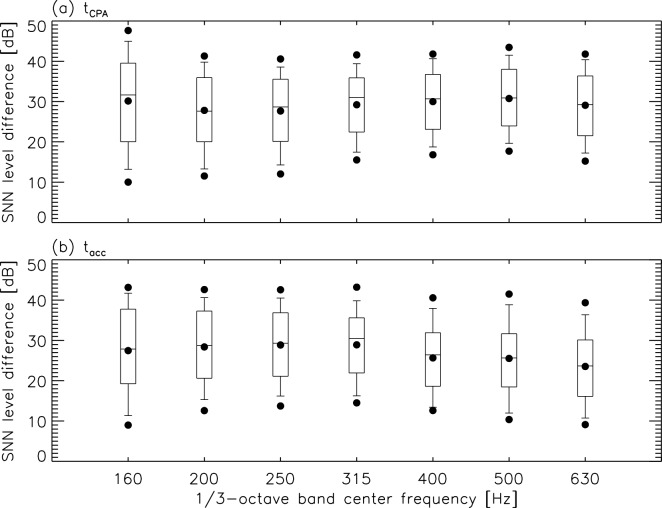
Whisker plots of the signal-plus-noise-to-noise (SNN) level difference ($\Delta {L}$) for all 1/3-octave bands used in this work (see Table 4). (**a**) $\Delta {L}$ distribution for signal recorded at $t_{\text {CPA}}$ (see Table 5). (**b**) $\Delta {L}$ distribution for signal recorded at $t_{acc}$ (see Table 5). Percentiles are shown, from bottom to top, at 5% (dot), 10%, 25%, 50%, 75%, 90%, and 95% (dot). The central dot gives the mean of the distribution.

## Appendix 1: Data processing following hydrophone retrieval

Local times are expressed with respect to the Eastern Daylight Time (EDT) zone (i.e., UTC-04:00). WAV files of the surrounding acoustic environment were recorded in continuous segments of 60 min, 12 times a day starting at the top of the hour, between 08:00:00 EDT and 19:59:59 EDT. Matlab$^{\circledR }$-supported PAMGuide^29^ was used to convert recorded WAV files into the frequency domain through power spectral density analysis by estimating the mean sound pressure levels (SPLs) in 1-second time windows from 1 to 144000 Hz. SPLs (in dB Hz$^{-1}$) were extracted using the Welch method^50^ with a Hann window^51^ and a 50% overlap, and will be hereafter referred to as received noise levels (RLs) at the hydrophone’s position. In order to match the temporal resolution of the AIS data, RLs spectra were averaged out into 1-min blocks providing 60 spectra for each 60-min WAV files, hence 720 spectra per day.

The water column height at the hydrophone’s position is regulated by the tidal height provided by the Fisheries and Oceans Canada’s 2020 Tide Table for Rivière-du-Loup (https://www.marees.gc.ca/fr/stations/3130). Time-dependent tidal heights, $\Delta {h}(t)$, were linearly interpolated over the 12-h time period of continuous recordings throughout the 46 days of deployment. The water column height at the hydrophone’s position is therefore defined as,

2 $$\begin{aligned} h(t)\,=\,h_{0}\,+\,\Delta {h}(t), \end{aligned}$$

where $h_{0}$ = 8 m (see “Acoustic data” section) and $\Delta {h}(t)$ is always positive.

According to Equation (1), the lowest frequency of interest is defined as,

3 $$\begin{aligned} f_{0}\,=\,\frac{c_{w,\text {RdL}}}{4h(t)_{\text {min}}\sqrt{1-(c_{w,\text {RdL}}/c_{b,\text {RdL}})^2}}, \end{aligned}$$

where $c_{w,\text {RdL}}$ = 1435 m s$^{-1}$ (Observatoire global du Saint-Laurent) and $c_{b,\text {RdL}}$ = 1500 m s$^{-1}$^32^ are approximations respectively of the water’s and seabed’s speeds of sound in the zone of interest of the Rivière-du-Loup harbor, and $h(t)_{\text {min}}$ is the lowest water column height at the hydrophone’s position recorded at the ferry’s closest-point-of-approach (CPA) during the 46 days of deployment. This was recorded at 08:11 EDT on the morning of 2020 July 23rd and was estimated at 8.43 m, hence giving 146 Hz from Equation (3). For the purpose of this work, $f_{0}$ was fixed at 141 Hz which corresponds to the lower boundary frequency ($f_{low}$) of the 1/3-octave band centered on 160 Hz (see Table 4).

**Table 4:** 1/3-Octave bands.

$f_{low}$ (Hz)	$f_{c}$ (Hz)	$f_{high}$ (Hz)
11	12	13
14	16	17
18	20	21
22	25	27
28	32	35
36	40	44
45	50	55
56	63	70
71	80	88
89	100	111
112	125	140
**141**	**160**	**177**
**178**	**200**	**223**
**224**	**250**	**281**
**282**	**315**	**354**
**355**	**400**	**446**
**447**	**500**	**561**
**562**	**630**	**707**

Bands used in this work are in bold.

At frequencies approaching 1000 Hz, computed transmission losses (see “Appendix 2”) often show a sudden increase that is not compensated by a substantial drop of the RLs obtained from the hydrophone’s recordings. This translated into source levels profiles increasing from mid-frequencies and above, a feature that does not agree with the monotonically decreasing behavior typically predicted by source levels models (see, e.g., Fig. 4 of^36^, Fig. 9 of^52^, Fig. 11 of^53^). This suggests that the noise signature detected above 1 kHz may not be attributed to the ferry itself but rather to a combination of non-laminar flow noise, instrumental self-noise and/or contamination of the fast-rotating engines of nearby pleasure crafts. Therefore, we used an upper cutoff frequency $f_{1}$ of 707 Hz which corresponds to the upper boundary frequency ($f_{high}$) of the 1/3-octave band centered on 630 Hz (see Table 4).

Note that further testing for mid-to-high frequencies (1000–100,000 Hz) using the better adapted ray tracing approach^54^ has confirmed that a very large proportion of the ferry’s acoustic power is indeed found at frequencies below $f_{1}$^15^. Extending the frequency domain above $f_{1}$ has no statistical impact on the conclusions reached in this work.

## Appendix 2: MSLs computation

All 60 of the averaged-out 1-min RLs spectra forming the 60-min time segments (see “Appendix 1”) in which CPA occurrences between the ferry and the hydrophone happened were spectrally collapsed between $f_{0}$ and $f_{1}$ to obtain the broadband BB$_{\text {RL}}$[$f_{0}-f_{1}$] measurement (in dB) for each minute of the corresponding hour. Hence,

4 $$\begin{aligned} \text {BB}_\text {RL}[f_{0}-f_{1}]\,=\,10.0\,\times \,\log _{10}\left( \sum _{i=f_{0}}^{f_{1}} 10^{\text {RL}_{i}/10} \right) , \end{aligned}$$

where *i* is the *i*th integer frequency between $f_{0}$ and $f_{1}$. Equation (4) allows to track in time the impact of the transiting ferry on the noise budget of the hydrophone’s surroundings.

Backpropagation, from the hydrophone’s location to the ferry’s position, was modeled using the split-step Padé approximation of the parabolic equation method^30^. The RAM algorithm was used to estimate the transmission loss (TL$_{\text {RAM}}$) due to the geometric sound attenuation sustained between the ferry (source) and the hydrophone’s position (receiver). According to the mooring design (see “Acoustic data” section), at each moment, the receiver’s depth, $z_{r}(t)$, is provided by,

5 $$\begin{aligned} z_{r}(t)\,\equiv \,h(t)\,-\,2.0, \end{aligned}$$

where the height *h*(*t*) of the water column at the hydrophone’s position is given by Equation (2). The source’s depth, $z_{s}$, was fixed at 2.94 meters according to ISO standard^11^, which represents 70% of the N.M. Trans Saint-Laurent draught of 4.2 m (see Table 1). Bathymetric data were retrieved from the Canadian Hydrographic Service and interpolated on a 100-m mesh grid. Sediment nature has been taken from the geological survey of^32^ which reveals a mixture of clay, silt and sand in our zone of interest. Geo-acoustical properties of the seabed were therefore approximated, according to Table 1.3 of^24^, to 1525 m s$^{-1}$, 1650 kg m$^{-3}$ and 0.7 dB $\lambda _{p}^{-1}$ respectively for the compressional speed of sound ($c_{b,\text {RdL}}$), density ($\rho _{b,\text {RdL}}$), and compressional wave attenuation ($\alpha _{p,\text {RdL}}$) in the sediments. Uncertainties on the sediments’ parameters along lines-of-sight connecting the ferry to the hydrophone may prevent from a direct comparison between the ferry’s underwater radiated noise and the noise radiated by other ship classes of the SLE. However, relative comparisons of the ferry’s radiated noise during different operational modes in the restrained area surrounding the hydrophone (*d* $\lesssim$ 1-2 km) are expected to hold.

Average water temperature and salinity profiles (with a 1-m resolution along the depth axis) are provided by the Observatoire global du Saint-Laurent close to our zone of interest (see Panels (a) and (b) of Fig. 9). In the figure’s Panel (c), the corresponding speed of sound value at depth *z*, $$c_{w}(z)$$, was provided by^55,]^$$^{\mathrm{Equation 2}}$$. A high-order polynomial fit was then applied to the resulting $$c_{w}(z)$$ data and coefficients were stored and later used to construct RAM input files (e.g., see Fig. 2 of^56^).

TL$_{\text {RAM}}$ was processed for each integer frequency between $f_{0}$ and $f_{1}$. Transmission loss due to magnesium sulfate and boric acid contributions (TL$_{\text {abs}}$) was treated according to the theory developed by^57,58^ with salinity and water acidity of 18$\text{\textperthousand}$ and 8 respectively. The water temperature at the time of the recording was provided by the output log file of the hydrophone for each 60-min block, ranging between $4.97\,^\circ \hbox {C}$ and $11.99\,^\circ \hbox {C}$ during the 46 days of data gathering.

Frequency-dependent source-level (SLs) spectra were processed using the passive SONAR equation,

6 $$\begin{aligned} \text {SL}_{i}(\phi ,\lambda )\,=\,\text {RL}_{i}(\phi _{0},\lambda _{0})\,+\,\text {TL}_{\text {RAM},i} (\phi ,\lambda \rightarrow \phi _{0},\lambda _{0})\,+\,\text {TL}_{\text {abs},i}(\phi ,\lambda \rightarrow \phi _{0},\lambda _{0}), \end{aligned}$$

where *i* is the *i*th frequency between $f_{0}$ and $f_{1}$, TL$_{\text {RAM},i}$($\phi$,$\lambda$ $\rightarrow$ $\phi _{0}$,$\lambda _{0}$) and TL$_{\text {abs},i}$($\phi$,$\lambda$ $\rightarrow$ $\phi _{0}$,$\lambda _{0}$) are respectively the geometric and absorption sound attenuation sustained between the ferry’s position ($\phi$,$\lambda$) and the hydrophone’s position ($\phi _{0}$,$\lambda _{0}$), and RL$_{i}$($\phi _{0}$,$\lambda _{0}$) and SL$_{i}$($\phi$,$\lambda$) are respectively the sound levels measured at the hydrophone and the monopole source levels computed at the ferry’s position.

BB$_{\text {MSL}}$[$f_{0}-f_{1}$] (in dB) is the broadband value of the frequency-integrated SLs spectrum between $f_{0}$ and $f_{1}$. Hence,

7 $$\begin{aligned} \text {BB}_{\text {MSL}}[f_{0}-f_{1}]\,=\,10.0\,\times \,\log _{10}\left( \sum _{i=f_{0}}^{f_{1}} 10^{\text {SL}_{i}/10} \right) , \end{aligned}$$

which allows to track in time noise radiated from the ferry’s position. Results at $t_{\text {CPA}}$ and $t_{acc}$ (see “Ferry’s MSL” section) are shown in Table 5.

**Table 5:** Data collected of the N.M. Trans-Saint-Laurent’s Transits during the 2020 SLE Summer Campaign.

Date	$t_{\text {CPA}}$ (EDT)	$d_{\text {CPA}}$ (km)	STW$_{\text {CPA}}$ (knots)	$a_{\text {CPA}}$ (knots min^−1^)	BB$_{\text {MSL}}^{\text {CPA}}$[$f_{0}-f_{1}$] (dB)	$t_{acc}$ (EDT)	$d_{acc}$ (km)	STW$_{acc}$ (knots)	$a_{acc}$ (knots min^−1^)	BB$_{\text {MSL}}^{acc}$[$f_{0}-f_{1}$] (dB)
2020-07-22	08:12	0.800	11.99	0.20	169.30	08:05	1.920	5.02	1.60	188.86
2020-07-22	12:12	0.776	11.58	− 0.05	164.47	12:05	1.928	4.97	1.46	177.03
2020-07-22	16:10	0.940	13.25	− 0.15	167.08	16:04	1.977	5.01	1.79	177.47
2020-07-23	08:11	0.828	13.20	− 0.01	177.46	–	–	–	–	–
2020-07-23	12:19	0.798	11.88	− 0.11	167.14	12:12	1.894	4.24	1.51	180.29
2020-07-23	16:14	0.934	13.49	− 0.15	171.37	16:08	1.875	5.04	1.78	183.34
2020-07-24	08:23	0.784	13.01	− 0.07	172.96	–	–	–	–	–
2020-07-24	12:22	0.988	13.22	0.06	169.23	12:15	2.038	5.39	1.34	176.07
2020-07-24	16:17	1.133	13.98	0.19	176.45	16:11	1.969	4.74	1.47	176.49
2020-07-25	08:17	0.540	13.37	− 0.05	169.92	08:10	1.945	5.63	1.19	185.65
2020-07-25	12:10	0.921	12.98	− 0.01	175.09	12:03	1.994	4.97	1.37	187.40
2020-07-25	16:10	1.011	13.67	− 0.17	171.04	16:03	2.037	4.76	1.36	174.53
2020-07-26	08:10	0.639	13.35	− 0.16	170.33	08:03	1.987	5.27	1.34	188.32
2020-07-26	12:15	0.988	12.73	− 0.10	177.36	12:07	1.967	5.29	1.26	186.52
2020-07-26	16:09	1.012	13.16	− 0.02	173.89	16:03	1.988	4.89	1.64	174.21
2020-07-27	08:12	0.858	13.87	− 0.13	172.89	08:06	1.989	4.98	1.71	178.98
2020-07-27	12:21	0.749	12.64	− 0.06	170.22	12:14	2.009	5.54	1.55	189.86
2020-07-27	16:11	0.867	12.63	− 0.10	168.86	16:04	2.003	4.64	1.52	172.27
2020-07-28	08:11	0.975	13.03	− 0.12	168.88	08:05	1.971	5.17	1.71	178.65
2020-07-28	12:16	0.788	12.05	− 0.12	167.78	12:09	1.969	5.73	1.62	187.57
2020-07-28	16:18	0.873	13.00	− 0.05	168.93	16:12	1.942	4.25	1.69	177.54
2020-07-29	08:15	0.937	13.04	0.11	164.95	08:09	1.938	5.54	1.71	178.77
2020-07-29	12:13	0.808	12.81	− 0.05	169.50	12:07	1.945	4.33	1.68	181.34
2020-07-29	16:11	0.931	12.66	0.06	167.01	16:04	2.043	4.14	1.45	176.21
2020-07-30	08:12	0.914	13.27	0.01	167.87	08:05	1.981	5.46	1.36	173.25
2020-07-30	12:17	0.877	13.36	− 0.03	176.54	12:10	2.002	4.42	1.52	182.86
2020-07-30	16:19	0.804	13.06	0.01	172.43	16:11	2.127	4.64	1.25	201.07
2020-07-31	08:10	0.914	13.37	− 0.01	173.93	08:03	2.000	4.13	1.64	177.59
2020-07-31	12:15	0.741	13.51	− 0.27	168.71	12:08	1.926	7.04	1.27	178.34
2020-07-31	16:09	0.953	13.08	0.02	173.30	16:02	2.042	5.47	1.44	183.78
2020-08-01	08:10	0.917	13.58	− 0.15	169.93	08:04	1.899	2.64	0.12	175.15
2020-08-01	12:14	1.185	13.84	0.17	178.51	12:08	2.007	5.79	1.44	180.48
2020-08-01	16:11	0.975	13.59	− 0.07	181.80	16:04	1.985	6.45	1.63	185.93
2020-08-02	08:11	0.916	13.54	− 0.17	175.35	08:04	2.028	6.43	1.43	183.29
2020-08-02	12:19	1.169	13.37	0.33	175.23	12:13	1.926	5.75	1.37	181.07
2020-08-02	16:11	0.962	13.68	− 0.09	176.76	16:05	1.906	5.78	1.87	180.87
2020-08-03	08:11	0.893	12.14	− 0.06	169.10	08:04	1.988	5.43	1.52	182.84
2020-08-03	12:22	0.904	12.98	− 0.01	168.11	12:16	1.889	5.51	1.54	180.06
2020-08-03	16:10	0.957	12.81	0.15	168.84	16:03	2.037	4.23	1.54	184.33
2020-08-04	08:12	0.788	13.17	0.03	176.73	–	–	–	–	–
2020-08-04	12:21	0.974	13.07	0.09	171.18	12:14	1.959	4.50	1.39	177.67
2020-08-04	16:17	0.827	13.85	− 0.06	174.96	16:11	1.985	5.04	1.71	185.07
2020-08-05	08:12	0.719	12.67	0.07	171.38	08:04	2.007	4.66	1.33	193.91
2020-08-05	12:31	0.803	11.17	− 0.06	169.66	12:23	1.893	4.57	1.15	176.39
2020-08-05	16:38	0.813	11.99	− 0.06	169.41	16:30	1.956	4.30	1.19	178.92
2020-08-06	08:13	0.797	11.88	− 0.17	173.04	08:05	1.976	6.11	1.18	192.78
2020-08-06	12:19	0.561	12.75	0.04	170.00	12:12	1.897	5.56	1.20	179.86
2020-08-06	16:11	0.828	13.53	0.26	174.10	16:04	1.910	7.19	1.02	183.12
2020-08-07	08:10	0.879	12.85	− 0.03	176.39	08:03	2.008	5.66	0.91	190.71
2020-08-07	12:21	1.043	13.43	− 0.21	169.28	12:14	2.043	5.94	1.36	174.17
2020-08-07	16:44	1.201	14.11	0.16	175.65	16:37	2.003	5.60	1.30	182.72
2020-08-08	08:16	0.908	13.15	− 0.07	174.53	08:09	1.969	6.95	1.30	186.50
2020-08-08	12:13	0.955	13.27	− 0.12	168.13	12:06	1.985	4.57	1.42	179.12
2020-08-08	16:12	0.838	13.75	0.14	172.41	16:05	1.941	4.70	1.15	178.50
2020-08-09	08:10	0.982	13.18	− 0.05	172.46	08:04	1.926	5.20	1.63	185.23
2020-08-09	12:10	0.869	12.86	− 0.13	172.39	12:03	1.979	5.23	1.48	181.56
2020-08-09	16:31	1.171	13.92	0.08	169.24	16:24	1.925	5.08	1.30	175.68
2020-08-10	08:13	0.788	13.70	0.06	172.15	08:07	1.969	4.71	1.79	185.17
2020-08-10	12:11	0.865	12.61	− 0.03	175.20	12:04	2.033	4.78	1.49	184.89
2020-08-10	16:13	0.831	13.46	0.04	170.65	16:07	1.966	5.43	1.61	175.65
2020-08-25	08:11	0.840	13.25	− 0.00	177.41	08:05	2.011	4.56	1.71	185.22
2020-08-25	12:12	0.820	12.09	0.03	175.59	12:04	2.066	4.14	1.47	184.62
2020-08-25	16:11	0.895	12.38	− 0.01	173.16	16:04	2.020	4.36	1.56	173.44
2020-08-26	08:11	0.969	13.16	− 0.14	170.53	08:05	1.980	4.03	1.85	181.69
2020-08-26	12:14	0.718	11.78	0.13	170.00	12:06	2.031	5.70	1.45	189.83
2020-08-26	16:11	0.815	12.80	− 0.08	175.75	16:04	1.956	5.03	1.61	180.77
2020-08-27	08:09	1.067	12.97	− 0.11	173.16	08:02	2.068	4.84	1.75	180.41
2020-08-27	12:11	1.014	12.91	− 0.09	173.29	12:04	2.023	5.18	1.45	186.59
2020-08-27	16:10	0.952	13.47	− 0.00	169.12	16:03	2.019	6.14	1.47	177.41
2020-08-28	08:18	1.132	13.21	− 0.00	174.21	08:11	1.972	4.88	1.46	176.49
2020-08-28	12:09	1.033	12.45	0.07	169.43	12:03	1.965	4.71	1.52	184.09
2020-08-28	16:14	0.852	13.00	− 0.02	176.78	16:07	1.997	5.51	1.77	183.07
2020-08-29	08:10	0.828	12.66	− 0.07	172.48	08:02	2.155	3.62	1.49	177.45
2020-08-29	12:09	1.032	13.32	− 0.07	168.93	12:03	1.909	5.40	1.79	180.30
2020-08-29	16:10	0.806	12.58	− 0.17	171.08	16:02	2.089	5.53	1.53	199.51
2020-08-31	08:11	0.889	12.72	− 0.09	176.96	08:04	1.997	4.45	1.57	183.78
2020-08-31	12:11	0.928	12.66	0.22	171.72	12:05	1.975	4.88	1.62	177.24
2020-08-31	16:12	0.728	13.31	− 0.08	172.90	16:07	1.730	4.42	1.42	182.48
2020-09-01	08:11	0.845	12.48	− 0.08	175.92	08:04	2.030	3.84	1.55	185.84
2020-09-01	12:11	0.867	13.78	− 0.16	177.59	12:05	1.849	5.46	1.75	178.69
2020-09-01	16:11	0.912	13.08	− 0.11	175.54	16:04	1.981	4.64	1.24	186.12
2020-09-02	08:11	0.655	12.77	0.12	169.93	08:04	1.975	4.86	1.61	188.82
2020-09-02	12:12	0.839	12.41	− 0.07	172.31	12:06	1.844	4.53	1.50	179.97
2020-09-02	16:11	0.823	12.82	− 0.06	174.04	16:05	1.948	4.27	1.64	187.00
2020-09-03	08:11	0.829	12.64	− 0.02	176.54	08:04	1.973	5.36	1.64	188.47
2020-09-03	12:14	0.940	12.84	− 0.09	168.97	12:07	2.005	5.21	1.33	174.90
2020-09-03	16:12	0.653	12.76	− 0.18	169.39	16:05	1.944	5.12	1.20	183.70
2020-09-04	08:11	0.897	12.32	0.03	175.67	08:04	2.016	5.20	1.24	194.28
2020-09-04	12:13	0.821	11.92	0.35	170.39	12:06	1.998	4.88	1.33	176.26
2020-09-04	16:11	0.520	13.05	0.00	166.52	16:03	2.002	5.18	0.99	179.05
2020-09-05	08:10	0.757	12.18	− 0.03	172.23	08:02	2.009	6.94	1.36	193.14
2020-09-05	12:11	1.029	12.18	− 0.05	170.64	12:03	2.050	5.65	0.96	174.12
2020-09-05	16:09	1.211	13.81	0.16	178.13	16:02	2.024	4.87	1.09	181.69
2020-07-22	11:08	0.770	13.46	0.29	172.65	11:14	1.862	5.19	− 1.36	181.00
2020-07-22	15:23	0.828	12.84	− 0.07	166.55	15:30	2.127	3.20	− 1.40	184.45
2020-07-22	18:42	0.868	13.12	− 0.21	168.36	18:47	1.834	3.48	− 1.40	192.36
2020-07-23	10:56	0.796	12.68	− 0.82	176.89	–	–	–	–	–
2020-07-23	15:23	0.832	13.54	− 0.19	170.21	15:29	2.093	3.83	− 1.70	180.63
2020-07-23	18:40	0.758	14.34	− 0.16	171.51	18:46	2.030	3.96	− 1.73	186.20
2020-07-24	11:20	0.867	14.14	− 0.55	176.33	11:25	1.738	4.43	− 1.39	191.28
2020-07-24	15:28	0.955	13.77	0.03	171.07	15:32	1.608	7.47	− 1.48	179.68
2020-07-24	19:05	0.900	13.89	− 0.06	175.50	19:10	1.872	5.61	− 1.87	187.27
2020-07-25	10:52	0.925	14.41	− 0.93	180.40	10:55	1.338	8.88	− 1.67	188.11
2020-07-25	15:18	0.806	12.83	0.04	167.00	15:23	1.818	6.74	− 1.54	178.86
2020-07-25	18:38	0.810	13.63	0.05	171.65	18:42	1.599	8.46	− 1.61	182.40
2020-07-26	10:47	0.825	14.93	− 0.13	175.60	10:53	2.013	3.56	− 1.15	195.31
2020-07-26	15:15	0.815	13.30	− 0.22	164.88	15:21	2.012	4.28	− 1.42	171.33
2020-07-26	18:45	0.843	12.63	− 0.24	167.54	18:51	2.024	3.99	− 1.43	186.60
2020-07-27	10:48	0.827	14.36	− 0.01	172.97	10:54	2.055	3.87	− 1.70	178.73
2020-07-27	15:05	0.825	13.36	− 0.47	166.55	15:11	2.012	4.47	− 1.37	172.08
2020-07-27	18:47	0.891	13.46	0.15	169.52	18:54	2.118	3.83	− 1.38	178.75
2020-07-28	10:48	0.818	13.30	− 0.20	165.74	10:52	1.731	6.96	− 1.82	184.36
2020-07-28	15:17	0.864	14.00	− 0.08	168.00	15:22	1.974	4.11	− 1.76	176.97
2020-07-28	18:50	0.841	13.03	0.24	170.17	18:56	1.889	5.05	− 1.48	182.38
2020-07-29	10:49	0.822	13.16	− 0.05	171.18	10:55	2.109	3.77	− 1.56	198.11
2020-07-29	15:04	0.817	13.21	− 1.05	172.85	15:07	1.384	7.33	− 1.48	183.37
2020-07-29	18:41	0.943	13.40	− 0.13	169.93	18:45	1.735	5.54	− 1.70	181.99
2020-07-30	11:00	0.690	14.16	− 0.10	174.51	11:08	2.075	3.79	− 1.32	180.77
2020-07-30	15:17	0.789	14.46	− 0.25	164.35	15:23	2.114	1.68	− 1.55	192.37
2020-07-30	18:42	0.882	13.90	− 0.69	169.73	18:48	2.007	3.95	− 1.18	175.82
2020-07-31	10:55	0.881	12.93	− 0.05	167.85	–	–	–	–	–
2020-07-31	15:09	0.841	14.20	0.01	178.62	15:12	1.396	8.95	− 1.67	186.34
2020-07-31	18:44	0.873	14.10	0.14	171.32	18:50	1.957	2.99	− 1.15	182.10
2020-08-01	10:58	0.843	12.56	− 0.06	163.78	–	–	–	–	–
2020-08-01	15:13	0.687	13.43	0.19	168.69	15:20	1.995	3.36	− 1.03	186.07
2020-08-01	18:29	0.865	14.11	− 1.25	170.79	18:33	1.733	6.48	− 1.64	187.89
2020-08-02	10:49	0.854	12.60	0.11	163.76	10:55	1.912	4.75	− 1.59	178.86
2020-08-02	15:10	0.827	13.92	− 0.07	172.46	15:16	2.063	4.35	− 1.62	185.85
2020-08-02	18:32	0.885	14.04	− 0.64	169.88	18:37	1.920	3.83	− 1.49	185.79
2020-08-03	11:17	0.834	13.96	− 0.04	165.03	11:22	1.930	5.71	− 1.75	175.14
2020-08-03	15:12	0.814	13.37	− 0.07	169.61	15:16	1.762	7.21	− 1.84	184.74
2020-08-03	18:36	0.785	14.57	− 0.81	171.76	18:41	1.820	5.13	− 1.48	192.68
2020-08-04	11:04	0.865	13.10	0.05	165.53	11:10	2.054	3.31	− 1.36	161.53
2020-08-04	15:30	0.885	12.98	0.13	170.60	15:36	2.079	3.23	− 1.61	183.11
2020-08-04	18:42	0.851	14.38	− 0.07	173.02	18:48	2.100	2.58	− 1.74	194.07
2020-08-05	11:18	0.903	13.61	− 0.43	170.81	11:23	1.692	5.20	− 1.33	181.58
2020-08-05	15:49	0.878	14.05	− 0.17	171.51	15:55	1.986	4.30	− 1.51	184.12
2020-08-05	19:10	0.813	14.57	− 0.43	173.34	19:16	1.991	2.70	− 1.32	189.02
2020-08-06	11:08	0.801	13.94	− 0.05	167.98	11:12	1.564	6.31	− 1.64	182.50
2020-08-06	15:21	0.862	13.35	− 0.91	166.49	15:28	2.096	3.81	− 1.31	181.65
2020-08-06	18:35	0.876	14.00	− 0.00	172.48	18:40	1.968	4.29	− 1.92	187.82
2020-08-07	11:17	0.829	14.02	− 1.15	171.67	11:19	1.218	11.14	− 1.48	180.09
2020-08-07	15:33	0.863	9.73	− 0.18	170.39	15:41	2.073	4.25	− 1.24	180.12
2020-08-07	19:33	0.818	12.70	− 0.09	166.05	19:39	2.001	4.13	− 1.44	187.04
2020-08-08	11:03	0.913	14.55	− 1.22	171.34	11:10	2.159	2.22	− 0.94	195.86
2020-08-08	15:20	0.806	12.10	0.00	167.09	15:23	1.283	9.35	− 1.09	178.08
2020-08-08	18:41	0.664	13.79	0.13	167.60	18:46	1.899	5.87	− 1.79	188.00
2020-08-09	10:50	0.858	14.26	− 0.48	171.40	10:53	1.352	8.05	− 1.59	181.67
2020-08-09	15:35	0.883	13.55	− 0.01	167.41	15:40	2.001	5.95	− 1.83	179.11
2020-08-09	19:22	0.838	13.86	− 0.16	171.37	19:26	1.665	8.12	− 1.66	181.76
2020-08-10	10:57	0.792	15.05	− 0.12	170.78	–	–	–	–	–
2020-08-10	15:14	0.894	13.13	0.05	168.00	15:18	1.693	10.19	− 1.80	179.51
2020-08-10	18:53	0.739	13.57	− 0.01	169.88	–	–	–	–	–
2020-08-25	10:36	0.861	13.84	− 1.30	173.97	10:41	1.951	4.46	− 1.52	185.07
2020-08-25	15:04	0.901	13.35	-0.99	172.13	15:06	1.181	10.42	− 1.28	175.71
2020-08-25	18:39	0.885	13.68	0.15	171.20	18:43	1.675	8.55	− 1.69	183.63
2020-08-26	10:44	0.865	13.93	− 0.02	172.31	10:52	2.168	1.71	− 0.77	191.84
2020-08-26	15:02	0.814	13.96	0.06	168.38	15:08	2.054	2.42	− 1.33	171.86
2020-08-26	18:55	0.822	13.31	0.13	168.47	–	–	–	–	–
2020-08-27	10:46	0.778	13.76	− 0.13	169.32	10:50	1.735	7.05	-1.86	186.28
2020-08-27	14:58	0.773	13.95	0.05	170.94	–	–	–	–	–
2020-08-27	18:38	0.833	13.95	0.00	167.55	18:43	1.943	6.34	− 1.64	180.47
2020-08-28	11:00	0.769	13.75	− 0.18	169.08	11:06	2.125	3.62	− 1.75	176.54
2020-08-28	14:59	0.841	14.28	0.00	172.43	–	–	–	–	–
2020-08-28	18:40	0.792	13.72	0.22	170.42	18:46	2.121	3.67	− 1.87	159.57
2020-08-29	10:46	0.791	13.07	− 0.40	167.49	10:52	2.009	4.68	− 1.50	183.74
2020-08-29	15:04	0.803	13.65	0.04	170.96	15:08	1.580	8.68	− 1.66	186.67
2020-08-29	18:37	0.885	13.29	− 2.00	170.89	18:39	1.290	9.50	− 1.41	179.08
2020-08-31	10:52	0.830	12.69	0.15	168.84	–	–	–	–	–
2020-08-31	15:09	0.838	13.19	− 0.09	170.42	15:15	1.995	4.31	− 1.70	181.04
2020-08-31	18:30	0.774	11.67	0.07	173.83	18:36	1.787	4.32	− 1.26	185.29
2020-09-01	10:49	0.877	12.51	0.23	169.46	10:54	1.824	7.12	− 1.52	176.34
2020-09-01	15:19	0.888	13.29	− 0.17	170.98	15:25	2.091	3.89	− 1.59	201.59
2020-09-01	18:32	0.861	14.14	− 0.70	176.39	18:38	1.922	2.95	− 1.31	189.90
2020-09-02	10:49	0.792	12.69	0.19	171.59	10:53	1.591	9.86	− 1.64	180.65
2020-09-02	15:10	0.782	13.07	0.10	170.72	15:16	2.073	4.91	− 1.84	177.32
2020-09-02	18:32	0.806	13.93	− 0.18	174.21	18:37	1.917	4.71	− 1.74	188.33
2020-09-03	10:50	0.897	12.37	0.11	167.40	10:54	1.559	6.96	− 1.41	185.17
2020-09-03	15:11	0.873	13.02	− 0.27	171.23	15:16	1.750	7.16	− 1.81	183.40
2020-09-03	18:36	0.825	13.61	0.03	179.05	18:40	1.518	7.56	− 1.61	190.28
2020-09-04	10:53	0.867	13.36	− 0.24	166.55	10:58	1.742	5.00	− 1.52	186.43
2020-09-04	15:15	0.867	12.90	− 1.77	170.15	15:17	1.208	8.92	− 1.27	171.34
2020-09-04	18:40	0.924	13.87	− 0.47	170.28	18:45	1.728	5.63	− 1.36	199.39
2020-09-05	10:46	0.857	13.21	0.09	168.60	10:50	1.539	6.89	− 1.52	182.83
2020-09-05	15:12	0.869	12.79	− 0.04	166.61	15:18	2.060	4.69	− 1.44	178.69
2020-09-05	18:39	0.858	14.00	− 0.95	173.28	18:45	1.939	4.41	− 1.19	189.00

## Appendix 3: Signal-to-noise ratio

Signal-plus-noise-to-noise level differences ($\Delta {L}$) at $t_{\text {CPA}}$ and $t_{acc}$ were computed for the 160, 200, 250, 315, 400, 500, and 630 Hz 1/3-octave bands (see Table 4) using the approach described in $\S$ 6.2 of^59^. In each case, the background profile was taken as the one spectrum with the lowest BB$_{\text {RL}}$[$f_{0}-f_{1}$] value in all 60 spectra forming the corresponding 60-min block. Recorded at a local time close to those of the time periods of interest (see Table 5), this background spectrum is likely representative of the weather conditions, sea activity, pleasure-craft density and flow noise contamination prevailing during the ferry’s transit. Whisker plots for $\Delta {L}$ are shown in Fig. 10. Background noise adjustment on RLs measurements was hence judged unnecessary because of $\Delta {L}$ values almost exclusively in excess of 10 dB (see Equation(4) of^59^).
